# Temporal Dynamics and Turnover of Rabbit Hemorrhagic Disease Virus 2 (RHDV2/GI.2) in Wild Lagomorphs from Northeastern Spain

**DOI:** 10.1007/s00248-026-02746-x

**Published:** 2026-03-21

**Authors:** Josep Estruch, Tereza Almeida, Emmanuel Serrano, Lorena Pereira, Carlos Rouco, Santiago Lavín, Joana Abrantes, Roser Velarde, Ana M. Lopes

**Affiliations:** 1 Wildlife Ecology & Health group (WE&H) and Servei d’Ecopatologia de Fauna Salvatge (SEFaS), Universitat Autònoma de Barcelona, Cerdanyola del Vallès, Barcelona, Spain; 2 CIBIO, Centro de Investigação em Biodiversidade e Recursos Genéticos, InBIO Laboratório Associado, Campus de Vairão, Universidade do Porto, Vairão, Portugal; 3 BIOPOLIS Program in Genomics, Biodiversity and Land Planning, CIBIO, Campus de Vairão, Vairão, Portugal; 4 Departamento de Biología Vegetal y Ecología, Universidad de Sevilla, Sevilla, Spain; 5 Departamento de Biologia, Faculdade de Ciências, Universidade do Porto, Porto, Portugal; 6 UMIB-Unit for Multidisciplinary Research in Biomedicine, ICBAS-School of Medicine and Biomedical Sciences, University of Porto, Porto, Portugal; 7 ITR, Laboratory for Integrative and Translational Research in Population Health, Porto, Portugal

**Keywords:** *Lepus europaeus*, *Oryctolagus cuniculus*, Rabbit hemorrhagic disease, Viral evolution, Viral recombination, Wildlife disease surveillance

## Abstract

In Mediterranean ecosystems, emerging lagoviruses, particularly rabbit hemorrhagic disease virus 2 (RHDV2/GI.2), have caused substantial mortality in European rabbits (*Oryctolagus cuniculus*) and, sporadically, hares (*Lepus* spp.). Recombination is common among RNA viruses, including lagoviruses, and shapes their evolution, influencing pathogenicity, epidemiology, and host range. However, the viral dynamics of recombinant strains in wild populations remain largely unknown. In this work, we analyse RHDV2/GI.2 coding sequences from European rabbits (*n* = 44), European brown hares (*Lepus europaeus*, *n* = 21) and one Iberian hare (*Lepus granatensis*) collected in Catalonia (NE Spain) between 2014 and 2024, to characterise temporal patterns and turnover of the different recombinant lineages. All strains shared the GI.2 capsid backbone but with four distinct non-structural genomic profiles: GI.3P–GI.2, GI.1bP–GI.2, GI.4(p16)–GI.1bP–GI.2, and GI.4P–GI.2. The four recombinant strains were detected in European brown hares, whereas rabbits were infected by only three, with the same recombinant present in areas where both species coexist. The Iberian hare was infected by a GI.4P-GI.2 recombinant, where rabbits showed the same lineage. Generalised additive models revealed significant interactions between recombinant type and year of detection, indicating contrasting turnover and temporal dynamics. Over the decade, GI.3P-GI.2 appeared transiently in 2014, GI.1bP-GI.2 strains dominated until 2019, and GI.4-related recombinants progressively replaced them, with GI.4P-GI.2 prevailing from 2020 onwards. Our findings suggest that recombinant strains may have been progressively replaced over time in leporid species from northeastern Iberia, involving variation in the non-structural genomic region that potentially contributes to differences in viral fitness, and supporting the role of recombination as a driver of viral dynamics and evolution.

**Supplementary Information:**

The online version contains supplementary material available at 10.1007/s00248-026-02746-x.

## Introduction

Emerging infectious diseases in wildlife can have profound ecological, economic, and conservation implications, particularly when they affect keystone species [1]. Viral disease outbreaks represent one of the most significant threats to wildlife populations due to viruses’ high mutation rates, ability to overcome host immunity, and cross-species transmission [2]. In recent decades, lagoviruses from the *Caliciviridae* family have gained significant attention due to their high virulence and capacity to cause extensive mortality in lagomorphs [3].

Rabbit hemorrhagic disease virus (RHDV/GI.1) and European brown hare syndrome virus (EBHSV/GII.1) have led to population declines, primarily in the European rabbit (*Oryctolagus cuniculus*) and the European brown hare (*Lepus europaeus*), respectively, across their distribution ranges since their first detection in the 1980s [4]. In 2010, a new lagovirus emerged in France [5], referred to as RHDV2 or GI.2, becoming a major concern due to its rapid global spread [6]. Both RHDV/GI.1 and RHDV2/GI.2 cause the rabbit hemorrhagic disease (RHD), which induces fulminant necrotic hepatitis in susceptible hosts [7, 8]. RHDV2/GI.2 has the potential to cross the species barrier, infecting *Lepus* species, other leporids, and possibly even other non-lagomorph species [9]. Several non-pathogenic lagoviruses from the GI and GII genogroups, traditionally referred to as rabbit and hare caliciviruses (RCV and HaCV, respectively), have been identified and detected in Europe and Australia [10–14]. These viruses, mainly enterotropic, are hypothesised to be the ancestors of pathogenic strains [15, 16].

Lagoviruses are small, non-enveloped and positive-sense RNA viruses with an icosahedral structure [8]. Their genome, of approximately 7.4 kb, is organised into two open reading frames (ORF) [17]. ORF1 encodes a large polyprotein subsequently cleaved into several non-structural proteins [p16, p23, a 2 C-like helicase, p29, VPg, a 3 C-like protease, and an RNA-dependent RNA polymerase (RdRp)], along with the major structural capsid protein VP60; ORF2 encodes the minor structural protein VP10. As it occurs with many other RNA viruses [18], multiple recombination events have been documented, with a recombination hotspot at the junction between the polymerase and the capsid [19]. In addition, occasional secondary recombination breakpoints have been identified at various locations throughout the genome [14, 20, 21]. Recombinant lagoviruses emerged through different combinations of pathogenic (GI.1, GI.2 and GII.1) and non-pathogenic (GI.3, GI.4 and GII.2) strains [19, 22–26]. These recombination events may influence viral characteristics, potentially enhancing pathogenicity, facilitating immune evasion, increasing resistance to vaccines, modifying cell or host tropism, or expanding the virus’ ability to infect new species [19, 27]. Recombinant lagoviruses follow a standardised nomenclature denoted as [RdRp genotype]P-[capsid genotype] [28].

Lagomorphs are keystone species in Mediterranean ecosystems, serving as the main prey for top predators such as the vulnerable Iberian lynx (*Lynx pardinus*) and Spanish imperial eagle (*Aquila adalberti*) [29]. Four lagomorph species inhabit the Iberian Peninsula, three of which occur in northeastern Spain: the European rabbit, the European brown hare, and the Iberian hare (*Lepus granatensis*) [30]. The emergence and spread of diseases affecting these species may not only impact their populations but also trigger cascading effects on predator communities and overall ecosystem stability [31]. Reports of RHDV2/GI.2 in European rabbits have been documented in the region since 2011, rapidly replacing RHDV/GI.1 strains [32, 33], as well as in European brown hares [34] and in the Iberian hare [35]. In European brown hares, RHDV2/GI.2 has been sporadically confirmed alongside with EBHSV/GII.1 cyclical outbreaks [36]. Characterisation of RHDV2/GI.2 circulating strains in the Iberian Peninsula revealed the emergence of multiple recombinants [20, 21, 37]; however, interspecies distribution patterns, as well as the spatiotemporal dynamics and turnover of these recombinants, remain poorly understood. In this study, we analysed RHDV2/GI.2 sequences from European rabbits (*n* = 44), European brown hares (*n* = 21) and one Iberian hare found dead between 2014 and 2024 throughout Catalonia (NE Spain). Our objectives were to (i) characterise the genetic diversity of circulating RHDV2/GI.2 strains in the three wild lagomorph species present in our study area, (ii) assess the multi-host occurrence of these recombinant strains, and (iii) investigate the temporal dynamics and turnover of RHDV2/GI.2 recombinant lineages in wild lagomorph populations from northeastern Spain.

## Materials and Methods

1. **Sample collection**

Samples used in this study, primarily liver (*n* = 64) and, when unavailable, spleen (*n* = 2), were collected from carcasses of 44 European rabbits, 21 European brown hares and one Iberian hare within the framework of the Wildlife Passive Surveillance Programme of Catalonia. Animals found dead in the field or displaying signs of disease are reported by hunters or residents to Rural Agents (Forestry Rangers of the Catalan Government), who are responsible for transporting the carcass to the Veterinary Faculty of the Autonomous University of Barcelona. This surveillance network covers the entire region of Catalonia and centralizes all post-mortem examinations of game species at the Veterinary Faculty. RHD is a listed disease for the World Organisation for Animal Health (WOAH) and, in Spain, notification of suspected and confirmed cases of RHD is also mandatory under national legislation (Royal Decree 779/2023). For this reason, suspected cases of RHDV2/GI.2, as determined by the macroscopic and microscopic lesions observed, were sent to regional (Laboratori de Sanitat Animal de Catalunya, LaSAC) and central (Laboratorio Central de Veterinaria de Algete) official laboratories for confirmation and notification of disease episodes. Confirmed RHDV2/GI.2 cases were finally analysed at the Research Centre in Biodiversity and Genetic Resources (CIBIO, Vairão, Portugal; see Sect.  2). In outbreaks where multiple carcasses were found together, only samples from the best-preserved individual were selected for molecular analysis.

2. **Genome amplification and identification of recombinants**

For molecular analyses, ~ 30 mg of liver/spleen tissue was homogenized in 300 µl of Lysis buffer (≈ 1:10 w/v) using a rotor-stator homogenizer (Mixer Mill MM400, Retsch) at 30 Hz for 7 min. Total RNA extraction and reverse transcription were performed using the GeneJet RNA Purification Kit (Thermo Scientific, Waltham, MA, USA) and the NZY First-Strand cDNA Synthesis Kit (NZYtech, Lisbon, Portugal), respectively, according to the manufacturer’s protocol.

Coding sequences were mainly obtained using a primer walking strategy [20]. Briefly, PCR reactions consisted of 1 µl of cDNA, 2 pmol of each primer, 5 µl of Phusion Flash High-Fidelity PCR Master Mix (Thermo Scientific, Waltham, MA, USA), and water to a final volume of 10 µl. PCR products were then purified and sequenced using the amplification primers on an ABI PRISM 3500 Genetic Analyzer (PE Applied Biosystems, Foster City, CA, USA). Primer pairs and PCR conditions are in Supplementary Table 1.

For samples where the primer walking strategy was unsuccessful (*n* = 14), high-throughput sequencing (HTS) was employed. RNA libraries were prepared using the NEBNext^®^ Ultra™ II RNA Library Prep Kit for Illumina^®^ in combination with the NEBNext rRNA Depletion Kit (New England Biolabs, USA), following the manufacturer’s protocol. The prepared libraries were sequenced at Macrogen Inc. (Seoul, South Korea) on an Illumina NovaSeq 6000 platform, generating 150 bp paired-end (PE) reads. Raw sequencing reads were assessed for quality using FastQC [38] and trimmed to remove low-quality bases and adapter sequences with Trimmomatic [39]. To eliminate host-derived sequences, filtered reads were aligned to the host reference genome (RefSeq, NCBI; rabbit GCF_964237555.1_mOryCun1.1 and Lepus GCF_033115175.1_mLepTim1.pri) using Bowtie2 [40] and matching reads were removed. The remaining non-host reads were subsequently mapped to the RHDV/GI.1 using Bowtie2, version 2.5.1 [40]. The newly obtained RHDV/GI.1 genome assembly for each sample was then used for downstream analyses. For those samples in which complete genomes could not be recovered by either the primer walking strategy or HTS (*n* = 15), sequences containing at least the known recombination breakpoints (p16 and RdRp-VP60 junction) were consistently obtained.

All the sequences produced are available in GenBank under accession numbers PX625220-PX625249, PX672492-PX672541 (*n* = 80), considering that for 15 samples there are two sequences per sample (p16 and RdRp-VP60 junction). The full sequence from the Iberian hare was already published in Velarde et al. [35], under accession number MZ203092. For genotype and recombinant assignment, the sequences were aligned with publicly available complete coding sequences representative of all major genetic groups [GI.1, GI.2, GI.3 and GI.4 (Supplementary Table 2)] and recombinant types using the BioEdit software, version 7.0.3 [41]. Then, maximum-likelihood phylogenetic trees were inferred separately for the capsid gene (VP60), RdRp, and a partial p16 region (nucleotides 130–438 relative to the RHDV/GI.1 reference sequence NC_001543). A total of 128 sequences were analysed [81 generated in this study and 47 retrieved from public databases (Supplementary Table 2)]. The best-fit nucleotide substitution model for each alignment was selected using ModelFinder implemented in IQ-TREE, applying the Bayesian Information Criterion, and corresponded to GTR + F+I+G4 for VP60, SYM + I+G4 for RdRp, and TIM2e+G4 for p16 [42]. Phylogenetic inference was conducted in IQ-TREE [43] with ultrafast bootstrap [44] using 1,000 replicates. Trees were midpoint-rooted, and branch lengths are proportional to the number of nucleotide substitutions per site.

3. **Statistical Analysis**

To assess the probability of recombinant occurrence over ten years of sampling, we fitted a generalised additive model (GAM) with a binomial error distribution and a logit link function [45]. The response variable was the infection status with one of the recombinant types for each hare or rabbit, while the interaction between the year of sampling (included as a smoothed term) and the recombinant type was used as the explanatory variable. Model selection was based on Akaike’s Information Criterion (AIC) [46]. Models with ∆i < 2 units have substantial support for explaining the observed variability in the variable of interest. Subsequently, we estimated the Akaike weight (Wi), defined as the relative likelihood of each model being the best among a set of candidate models [46]. Once the best model was selected, we evaluated the fit of GAM, checking the Un-Biased Risk Estimator (UBRE) and the overdispersion parameter [47].

## Results

**Fig. 1:**
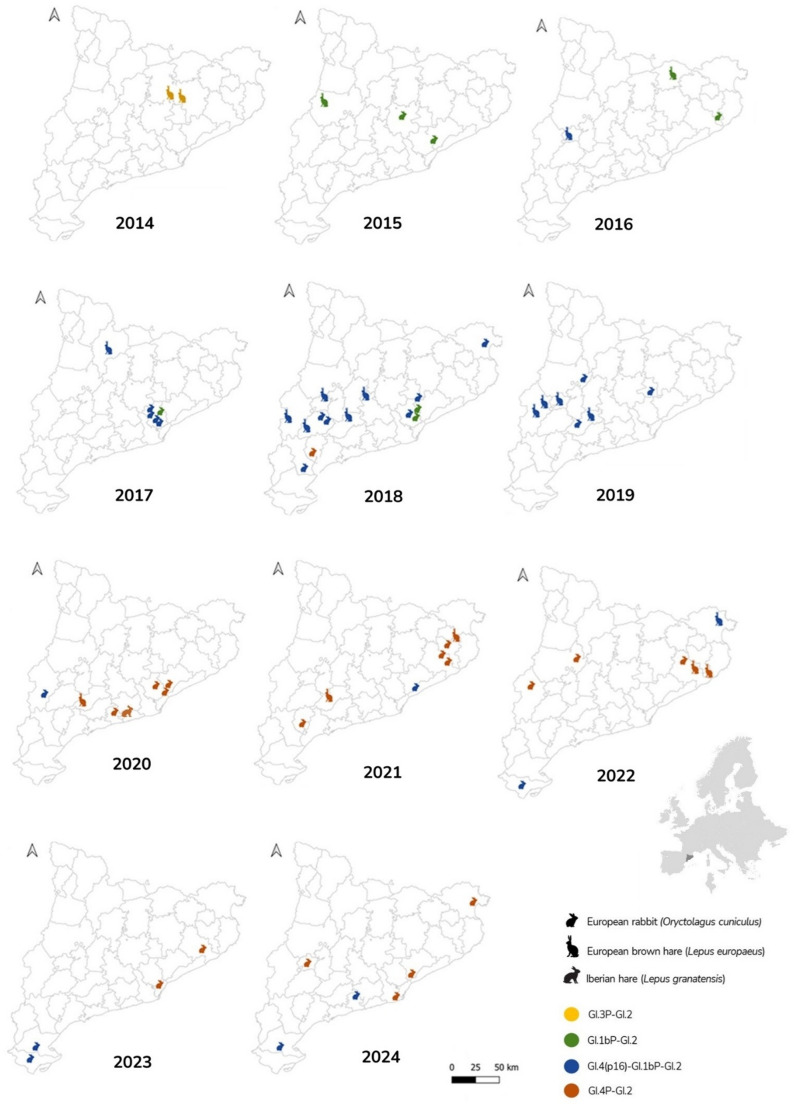
Spatial distribution of the different RHDV2/GI.2 recombinants in European rabbits (*Oryctolagus cuniculus*), European brown hares (*Lepus europaeus*) and Iberian hare (*Lepus granatensis*) over the years (2014–2024) in Catalonia, northeastern Spain

Complete or partial coding sequences from 66 RHDV2/GI.2-infected wild lagomorphs sampled in Catalonia (NE Spain) were successfully recovered, including European rabbits, European brown hares, and one Iberian hare (Supplementary Table 3). Following the standardised phylogenetically-based nomenclature proposed by Le Pendu et al. [28], in European brown hares, we identified four different recombinant strains: GI.3P-GI.2 (*n* = 2), GI.1bP-GI.2 (*n* = 2), GI.4P-GI.2 (*n* = 5) and GI.4(p16)-GI.1bP-GI.2 (*n* = 12). In European rabbits, we detected GI.1bP-GI.2 (*n* = 6), GI.4P-GI.2 (*n* = 18) and GI.4(p16)-GI.1bP-GI.2 (*n* = 20) (Supplementary Fig. 1). The sequence obtained from the Iberian hare corresponded to GI.4P-GI.2 [35] and is included in the present study for comparative analyses. The spatiotemporal distribution of cases among the three species is presented in Fig. 1. Although in-depth analyses were not performed due to the opportunistic nature of the data, no clear spatial relationships were observed, as all recombinants were detected across different regions of Catalonia, occasionally circulating simultaneously in nearby areas (Fig. 1; years 2017 and 2018). Regarding the species distribution of the different lineages, in areas where several cases in different species were confirmed, both rabbits and hares were infected by the same recombinant (Fig. 1).

To infer the probability of the different recombinants’ occurrence and understand possible temporal evolution patterns, we constructed and analysed diverse GAM models. According to our model selection, the best model explaining the occurrence of the different recombinant types included the interaction between the recombinant and the year of sampling (Table 1). The second-ranked model, which only included the recombinant type, was at 57.61 units of ∆I and thus had little support to explain the RHDV2/GI.2 recombinant dynamics in our study area. The interaction model explained 42.3% of the observed variability in the probability of detecting one of the four recombinants. This model has a UBRE of -0.26 and an overdispersion parameter lower than one, indicating a good fit for the data. The interaction with the year of sampling was significant for the four recombinant types except for GI.3P-GI.2 (year * GI.3P-GI.2, Chisq = 7.69, p-value = 0.05; GI.1bP-GI.2, Chisq = 14.55, p-value < 0.001; year * GI.4P-GI.2, Chisq = 41.45, p-value < 0.001, and year * GI.4(p16)-GI.1bP-GI.2, Chisq = 37.99, p-value < 0.001).

**Table 1:** Model selection of five generalized additive models (GAM), exploring the temporal trend in the likelihood of four lagoviruses’ recombinant types detected in European rabbits (*Oryctolagus cuniculus*; *n* = 44), European brown hares (*Lepus europaeus*; *n* = 21) and Iberian hare (*Lepus* granatensis; *n* = 1), collected in Catalonia, northeast Spain. k = number of estimated parameters, AIC = Akaike’s Information Criterion, Δi **=** difference of AIC between each model and the most parsimonious one, Wi = Akaike’s weight of the model. In bold is the selected model

Biological Models	k	AIC	Δi	Wi
Year * Recombinant type	12	195.03	0.00	1
Recombinant type	4	252.64	57.61	0
Year	5	254.63	59.61	0
Year + Recombinant type	5	254.63	59.61	0
Mo	1	298.91	103.88	0

**Fig. 2:**
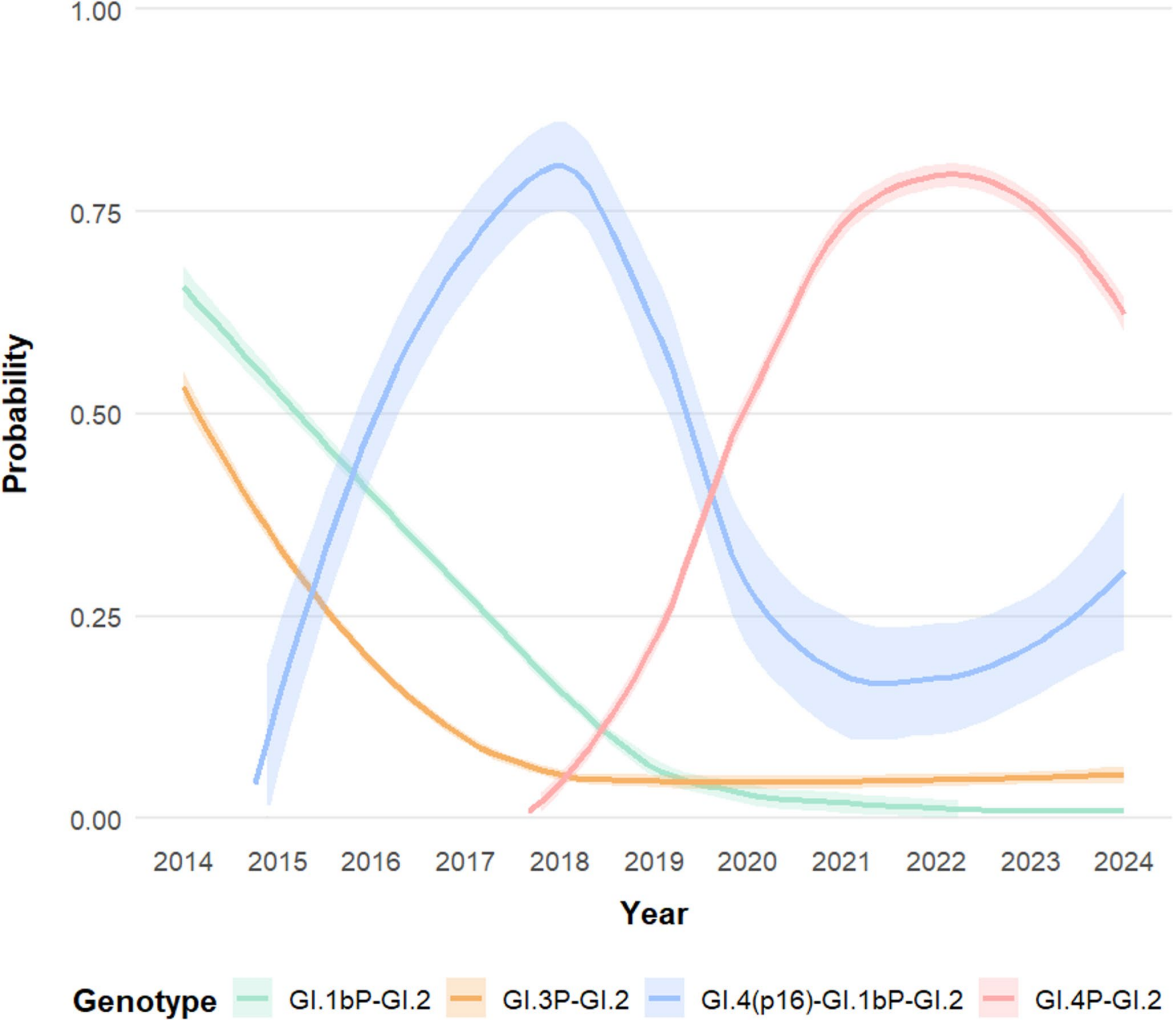
Temporal dynamics in the probability of occurrence of the different RHDV2/GI.2 recombinants circulating in European rabbits (*Oryctolagus cuniculus*), European brown hares (*Lepus europaeus*) and the Iberian hare (*Lepus granatensis*) between 2014 and 2024 in Catalonia (northeastern Spain). Values have been estimated using a generalized additive model (GAM)

Between 2014 and 2024, the temporal distribution of RHDV2/GI.2 recombinants showed a clear progression of dominant strains (Figs. 1 and 2). In 2014, the earliest recombinants detected were GI.3P-GI.2 (*n* = 2), although they were not detected in subsequent years. By 2015, GI.1bP-GI.2 emerged, persisting through 2018 alongside the appearance of GI.4(p16)-GI.1bP-GI.2 recombinants (first detected in 2016). From 2017 to 2019, GI.4(p16)-GI.1bP-GI.2 became increasingly frequent, accounting for the majority of recombinant detections each year (2017: *n* = 5; 2018: *n* = 11; 2019: *n* = 7), while GI.1bP-GI.2 continued to be detected at lower frequencies. Starting in 2020, GI.4P-GI.2 recombinants appeared and rapidly became the predominant strains until 2024, with GI.4(p16)-GI.1bP-GI.2 still detected sporadically (2020–2024: *n* = 1–2 per year).

## Discussion

In our study, we detected and traced the spatiotemporal dispersal patterns of RHDV2/GI.2 recombinants circulating in wild lagomorphs and that were previously reported in other regions of Spain, Portugal, and France [20, 21, 48]. These recombinants primarily involved GI.3, GI.1b and GI.4 as donors of the entire non-structural genome region, but also configurations where GI.4 contributed to the p16 and GI.1b to the remaining non-structural proteins, resulting in triple recombinants. These recombinants were all detected in European rabbits and European brown hares, except for GI.3P-GI.2, which was detected only in two hares in 2014. GI.3P-GI.2 is considered the original RHDV2/GI.2 virus, itself a recombinant [19, 49], which was first reported in France in 2010. This strain was also shown to be circulating among rabbits at the time of its emergence in Spain in 2011, near our study area, in Navarra [32]. Thus, the absence of GI.3P-GI.2 detections in rabbits in 2014 in our study is likely attributable to sampling bias, as no rabbits were collected for post-mortem analyses within the passive disease surveillance that year.

Based on the spatial distribution of infected individuals and the types of recombinants identified, there is no evidence of species-specific RHDV2/GI.2 lineages. In most areas, hares infected with a given recombinant were found near rabbits carrying the same recombinant, indicating overlap in viral circulation. These observations, together with findings from previous studies, suggest that RHDV2/GI.2 recombinants primarily circulate in rabbits and only occasionally spill over into sympatric hare populations [27, 34, 35, 50–52]. Interestingly, despite the co-circulation of EBHSV/GII.1 in our region and the frequent coexistence of hares with dense rabbit populations [36], inter-genogroup recombination has not been observed, in contrast with results from Germany and Italy [23, 26]. The underlying reason for this absence remains unclear; it might simply reflect stochastic processes, or it may suggest that, even when interspecific interactions occur, additional conditions are required for successful intergenogroup recombination.

The temporal evolution of RHDV2/GI.2 recombinants in the northeastern Iberian Peninsula may reveal a dynamic pattern, characterized by the emergence, dominance, and replacement of distinct lineages over time, with each recombinant having a particular temporal course. The GI.3P–GI.2 recombinant was detected exclusively in 2014, with no evidence of sustained circulation thereafter, despite its detection in other countries the subsequent years [53–55]. Other studies likewise did not detect this recombinant in the Iberian Peninsula between 2014 and 2016. This suggests it was possibly replaced by other recombinants with an increased viral fitness [20, 21], most likely by GI.1bP–GI.2, which, according to our data, circulated at least between 2015 and 2018. The circulation of the parental RHDV/GI.1b was historically restricted to the Iberian Peninsula [56, 57], making the emergence of GI.1bP–GI.2 there unsurprising while both GI.1b and GI.3P-GI.2 were still co-circulating.

Interestingly, from 2016 onwards, recombinants incorporating GI.4 non-structural genes began to emerge and progressively became dominant, suggesting that the GI.4 genetic background may be associated with increased epidemiological success, although the underlying mechanisms cannot be inferred from the present data. A similar phenomenon appears to have occurred in some regions of Australia, where genomic analyses showed that RHDV2/GI.2 structural genes combined with GI.4 non-structural genes rapidly replaced the previously dominant GI.1bP–GI.2 recombinant strains. This shift took place despite no detectable antigenic changes in the capsid, leading the authors to propose that non-structural proteins may play a pivotal role in epidemiological fitness [27]. In our study, although present at less frequency in more recent years, GI.4(p16)–GI.1bP–GI.2 recombinants have been co-circulating with GI.4P-GI.2. While different lineages can coexist at relative equilibrium over extended periods, as observed in Australia [27, 58], this finding suggests that the GI.4-derived p16, even with a GI.1b donor for the other non-structural proteins, may also outcompete other recombinants, thereby explaining its continued persistence. The precise role of the p16 remains unclear [59], despite mutations in the corresponding coding region have been linked to changes in virulence [60]. In other caliciviruses, such as noroviruses, non-structural proteins located at the N-terminal region have been proposed to act as an enhancer of immune evasion [61, 62]. Hence, recombination not only contributes to lagovirus genetic diversity but also introduces variation in non-structural genomic regions that may influence epidemiological dynamics.

Passive health monitoring plays a crucial role in wildlife health research, providing valuable data that forms the foundation for a robust epidemiological perspective [63]. Nonetheless, this study has some inherent limitations that should be considered. The number of genomes analysed per year varied due to the opportunistic nature of passive surveillance in wild populations, and in some years, only a few individuals were available. Differences in abundance, distribution, and public perception between rabbit and hare species may influence carcass collection and, consequently, the representativeness of passive surveillance data, particularly for the geographically restricted Iberian hare, located in just a few areas from southern Catalonia. Additionally, the study focused on wild lagomorphs and did not include domestic or farmed rabbits, which could provide complementary information on RHDV2/GI.2 dynamics. Finally, our findings are based on genomic and epidemiological data, without direct experimental evidence on viral characteristics or host responses.

Despite these constraints, the longitudinal scope of the dataset and the genomic resolution achieved in our study provide a solid framework for interpreting recombinant diversity and circulation dynamics over time in northeastern Spain. The overlapping spread of recombinants in rabbits and hares, combined with the known capacity of RHDV2/GI.2 to infect multiple leporid and non-lagomorph hosts, highlights the importance of multi-host systems in facilitating viral exchange and the emergence of novel variants. Continuous genomic surveillance, particularly in areas where multiple susceptible species coexist, will be essential to detect new recombinants at an early stage, assess their epidemiological potential, and anticipate their impact on wildlife populations and ecosystem integrity.

## Supplementary Information

Below is the link to the electronic supplementary material.

Supplementary Material 1

## Data Availability

The datasets analysed during the current study are available in the GenBank repository under accession numbers PX625220-PX625249 and PX672492-PX672541.
