# Supplementary material for: Temporal Dynamics and Turnover of Rabbit Hemorrhagic Disease Virus 2 (RHDV2/GI.2) in Wild Lagomorphs from Northeastern Spain
Source: Microb Ecol. 2026 Mar 21;89(1):89. doi: 10.1007/s00248-026-02746-x (PMC13053380; doi:10.1007/s00248-026-02746-x)
Supplement: Supplementary file 1 — Supplementary Material 1 [file 248_2026_2746_MOESM1_ESM.docx]

**Temporal dynamics and turnover of rabbit hemorrhagic disease virus 2 (RHDV2/GI.2) in wild lagomorphs from northeastern Spain**

Josep Estruch^1*^, Tereza Almeida^2,3^, Lorena Pereira^2,3^, Emmanuel Serrano^1^, Carlos Rouco^4^, Santiago Lavín^1^, Joana Abrantes^2,3,5^, Roser Velarde^1*^ and Ana M. Lopes^6,7^

^1^ Wildlife Ecology & Health group (WE&H) and Servei d’Ecopatologia de Fauna Salvatge (SEFaS), Universitat Autònoma de Barcelona, Barcelona, Spain.

^2^CIBIO, Centro de Investigação em Biodiversidade e Recursos Genéticos, InBIO Laboratório Associado, Campus de Vairão, Universidade do Porto, Vairão, Portugal.

^3^BIOPOLIS Program in Genomics, Biodiversity and Land Planning, CIBIO, Campus de Vairão, Vairão, Portugal.

^4^Departamento de Biología Vegetal y Ecología, Universidad de Sevilla, Sevilla, Spain

^5^Departamento de Biologia, Faculdade de Ciências, Universidade do Porto, Porto, Portugal.

^6^UMIB-Unit for Multidisciplinary Research in Biomedicine, ICBAS-School of Medicine and Biomedical Sciences, University of Porto, Porto, Portugal.

^7^ITR, Laboratory for Integrative and Translational Research in Population Health, Porto, Portugal.

*Corresponding authors: [josep.estruch@uab.cat](mailto:josep.estruch@uab.cat) (JE), [roser.velarde@uab.cat](mailto:roser.velarde@uab.cat) (RV), [jabrantes@cibio.up.pt](mailto:jabrantes@cibio.up.pt) (JA)

**Supplementary Figures:**

**
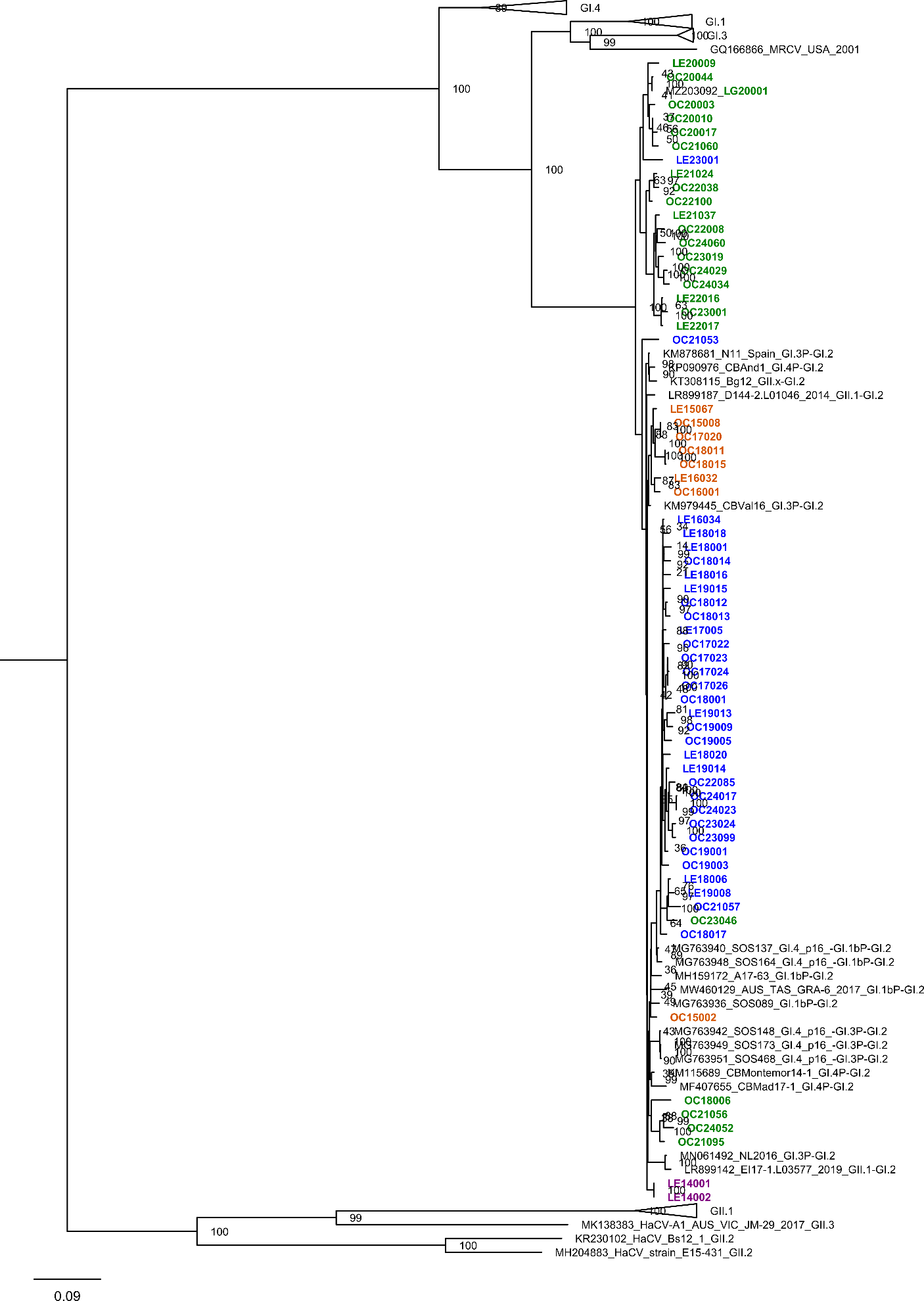
A)**

**
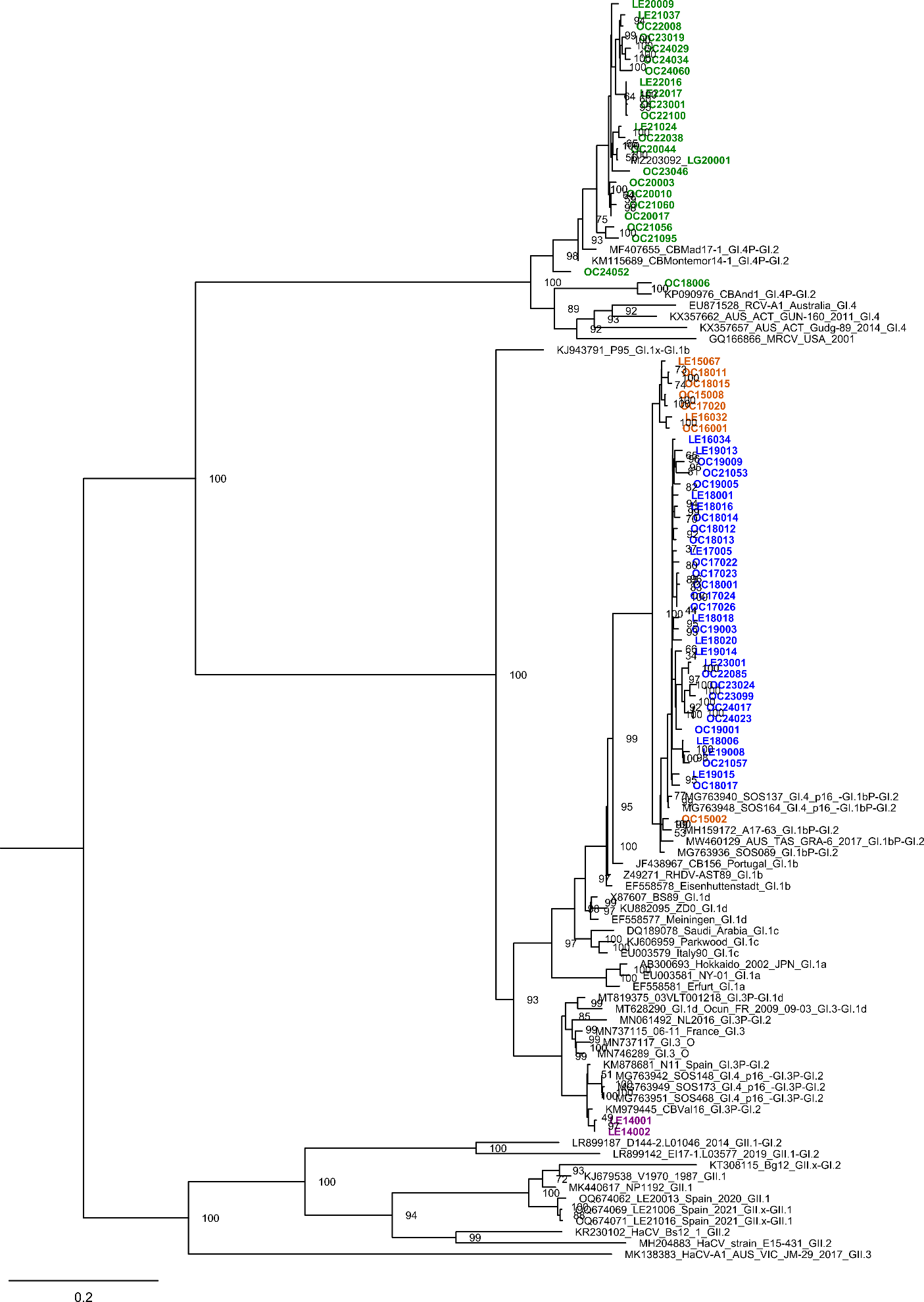
B)**
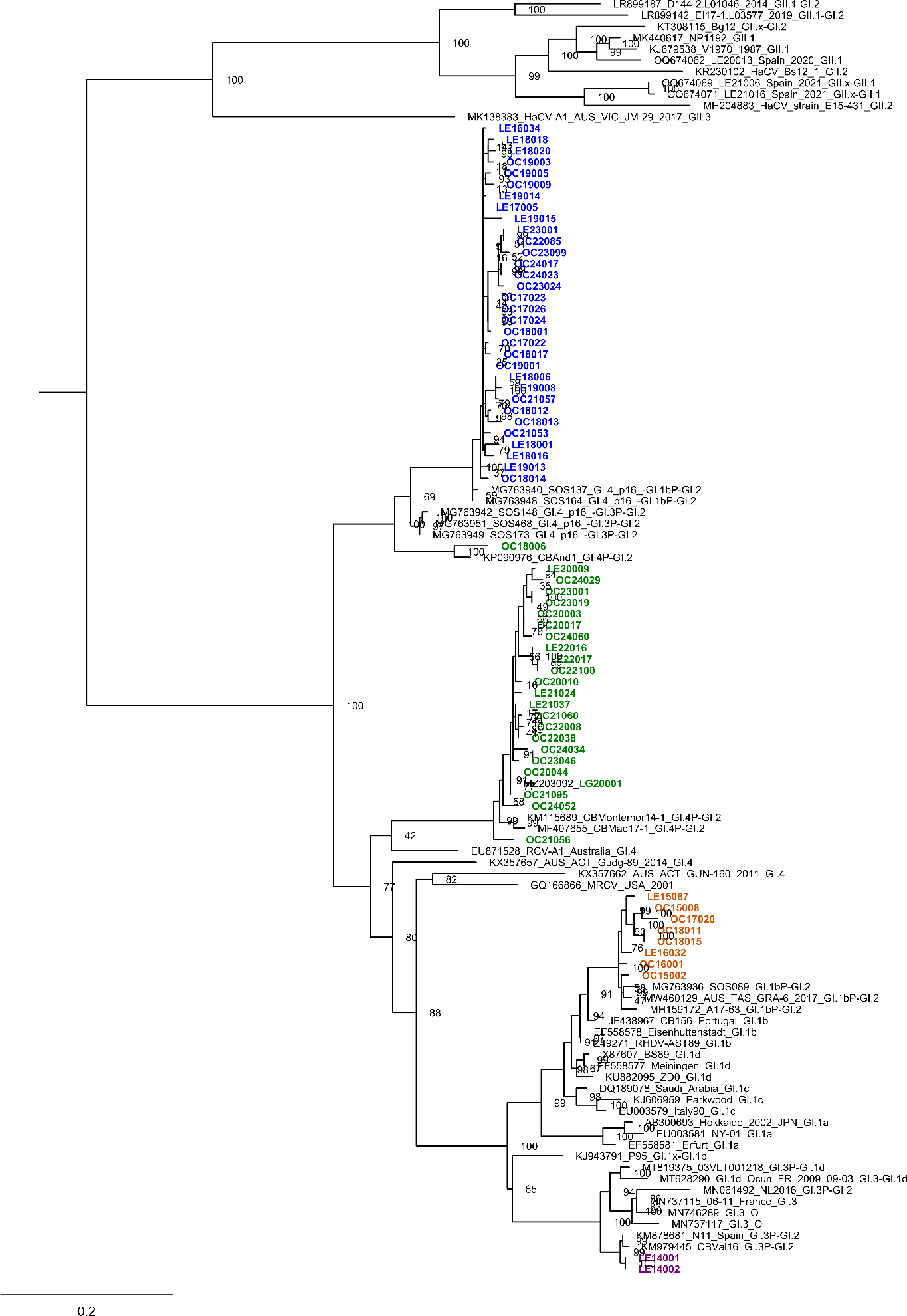


**C)**

**Supplementary Fig. 1.** Maximum-likelihood phylogenetic trees inferred for (A) VP60, (B) RdRp and (C) partial p16. GI.3P–GI.2 strains identified in this study are shown in purple, GI.1bP–GI.2 in orange, GI.4(p16)–GI.1bP–GI.2 in blue, and GI.4P–GI.2 in green. GenBank accession numbers for reference sequences are indicated and listed in Supplementary Table 2.

**Supplementary Table 1.** PCR primers and amplification conditions.

| Primer name | Sequence (5’ – 3’) | Annealing temperature (ºC) | Extension time (s) | Product length (bp) |
| --- | --- | --- | --- | --- |
| RHDV0078F  RHDV1530R | CTTCCTGGACCTCAGGGACAAG  CATGACGAAGCAGAGACCAC | 52 | 50 | 1452 |
| RHDV0078F  RHDVb460RC | CTTCCTGGACCTCAGGGACAAG  CCGAGCTCAATAAACAAGTC | 53 | 50 | 382 |
| RHDV0087F  RHDVb460RC | CAAGACCCCTCCCTGTTGT  CCGAGCTCAATAAACAAGTC | 53 | 6 | 373 |
| RHDV1432F  RHDV2515R | AGGTGCACCCTGCCATCATACAA  TTCCACTCACAGACCACGCTCAT | 54 | 16 | 1083 |
| RHDV2356F  RHDV3592R | CAACATCTTTGGCGCATGGT  AAGATTGACGTGCTGGCGTA | 56 | 19 | 1236 |
| RHDV4584F  RHDVb5400RC | AGCAAACGGAGCTCACCAA  GGTGTTGTGGCCACTACTAG | 52 | 13 | 816 |
| RHDV5302F  RHDV6282R | GTACCTGACGACGAATTTGTGAATG  CTGGAGCAATTTGGGAGATG | 56 | 15 | 980 |
| RHDV6186F  RHDV6748R | CATTGACCACGACAGAGGTAAC  CTGAGGCCGGTTCAACTAACG | 67 | 8 | 562 |
| RHDV6661F  RHDV7437R | GCCGCTCCTATTGGCAAGAAC  TTGGGTTTATAGTTTAAAGTAAGCTAT | 53 | 10 | 776 |

**Supplementary Table 2.** Representative sequences of the different genotype lineages and recombinant types of lagoviruses used in the alignment, including GI.1-4 and GII.1-3. Recombinant lagoviruses follow a standardized nomenclature denoted as [RdRp genotype]P-[capsid genotype] [28].

| Sequence ID | Genotype/variant | Recombinant type | Country | Year | GenBank Accession | Reference |
| --- | --- | --- | --- | --- | --- | --- |
| Hokkaido/2002/JPN | GI.1a | - | Japan | 2002 | AB300693 | [64] |
| NY-01 | GI.1a | - | USA | 2001 | EU003581 | [65] |
| Erfurt | GI.1a | - | Germany | 2000 | EF558581 | [66] |
| Eisenhuttenstadt | GI.1b | - | Germany | 1989 | EF558578 | [66] |
| CB156 | GI.1b | - | Portugal | 1997 | JF438967 | [67] |
| RHDV-AST89 | GI.1b | - | Spain | 1989 | Z49271 | [68] |
| P95 | GI.1b | GI.xP-GI.1b | Portugal | 1996 | KJ943791 | [69] |
| Saudi_Arabia | GI.1c | - | Saudi Arabia | 2006 | DQ189078 | [70] |
| Parkwood | GI.1c | - | Australia | 1998 | KJ606959 | [60] |
| Italy90 | GI.1c | - | Italy | 1990 | EU003579 | [65] |
| BS89 | GI.1d | - | Italy | 1989 | X87607 | Rossi 1995 (Unpublished) |
| Meiningen | GI.1d | - | Germany | 1993 | EF558577 | [66] |
| ZD0 | GI.1d | - | Poland | 2000 | KU882095 | Fitzner 2016 (Unpublished) |
| 03VLT001218 | GI.1d | GI.3P-GI.1d | Sweden | 2003 | MT819375 | [49] |
| GI.1d/Ocun/FR/2009/09-03 | GI.1d | GI.3P-GI.1d | France | 2009 | MT628290 | [49] |
| N11_Spain | GI.2 | GI.3P-GI.2 | Spain | 2011 | KM878681 | [37] |
| CBVal16 | GI.2 | GI.3P-GI.2 | Portugal | 2012 | KM979445 | [37] |
| NL2016 | GI.2 | GI.3P-GI.2 | Netherlands | 2016 | MN061492 | [71] |
| SOS148 | GI.2 | GI.4 (p16)- GI.3P-GI.2 | Portugal | 2015 | MG763942 | [19] |
| SOS173 | GI.2 | GI.4 (p16)- GI.3P-GI.2 | Portugal | 2015 | MG763949 | [21] |
| SOS468 | GI.2 | GI.4 (p16)- GI.3P-GI.2 | Portugal | 2015 | MG763951 | [21] |
| SOS089 | GI.2 | GI.1bP-GI.2 | Portugal | 2014 | MG763936 | [21] |
| A17-63 | GI.2 | GI.1bP-GI.2 | Morocco | 2017 | MH159172 | [22] |
| AUS/TAS/GRA-6/2017 | GI.2 | GI.1bP-GI.2 | Australia | 2017 | MW460129 | [27] |
| SOS137 | GI.2 | GI.4(p16)-GI.1bP-GI.2 | Portugal | 2015 | MG763940 | [21] |
| SOS164 | GI.2 | GI.4(p16)-GI.1bP-GI.2 | Portugal | 2015 | MG763948 | [21] |
| CBAnd1 | GI.2 | GI.4P-GI.2 | Spain | 2012 | KP090976 | [20] |
| CBMontemor14-1 | GI.2 | GI.4P-GI.2 | Portugal | 2014 | KM115689 | [20] |
| CBMad17-1 | GI.2 | GI.4P-GI.2 | Portugal (Madeira) | 2017 | MF407655 | [69] |
| D144-2.L01046/GER-NW/2014 | GI.2 | GII.1P-GI.2 | Germany | 2014 | LR899187 | [23] |
| EI17-1.L03577/GER-NW/2019 | GI.2 | GII.1P-GI.2 | Germany | 2019 | LR899142 | [23] |
| Bg12 | GI.2 | GII.x-GI.2 | Italy | 2012 | KT308115 | [34] |
| 06-11_France | GI.3 | - | France | 2006 | MN737115 | [19] |
| GI.3/O cun/FR/2009/09-100 | GI.3 | - | France | 2009 | MN737117 | [19] |
| GI.3/O cun/FR/2008/08-133 | GI.3 | - | France | 2009 | MN746289 | [19] |
| RCV-A1_Australia | GI.4 | - | Australia | 2007 | EU871528 | [73] |
| AUS/ACT/Gudg-89/2014 | GI.4 | - | Australia | 2014 | KX357657 | [74] |
| AUS/ACT/GUN-160/2011 | GI.4 | - | Australia | 2011 | KX357662 | [74] |
| MRCV | - | - | USA | 2001 | GQ166866 | [75] |
| NP1192 | GII.1 | - | Poland | 1992 | MK440617 | Fitzner et al. 2019 (Unpublished) |
| V1970 | GII.1 | - | Sweden | 1987 | KJ679538 | [76] |
| LE20013_Spain_2020 | GII.1 | - | Spain | 2020 | OQ674062 | [25] |
| LE21006_Spain_2021 | GII.1 | GII.2(p16+p23)-GII.1 | Spain | 2021 | OQ674069 | [25] |
| LE21016_Spain_2021 | GII.1 | GII.2(p16+p23)-GII.1 | Spain | 2021 | OQ674071 | [25] |
| Bs12_1 | GII.2 | - | Italy | 2012 | KR230102 | Cavadini et al. 2015 (Unpublished) |
| E15-431 | GII.2 | - | France | 2015 | MH204883 | [77] |
| HaCV-A1/AUS/VIC/JM-29/2017 | GII.3 | - | Australia | 2017 | MK138383 | [14] |

**Supplementary Table 3.** General metadata of the 81 sequences analysed in this study, obtained from the 66 RHDV2/GI.2-infected lagomorphs in Catalonia (NE Spain), including 44 European rabbits (*Oryctolagus cuniculus*), 21 European brown hares (*Lepus europaeus*) and one Iberian hare (*Lepus granatensis*).

| Sample ID | GenBank Accession | Host species | Municipality | Collection date | Tissue | Strain |
| --- | --- | --- | --- | --- | --- | --- |
| LE14001 | PX625220, PX625247 | *Lepus europaeus* | Oristà | 13-Feb-14 | Liver | GI.3P-GI.2 |
| LE14002 | PX672492 | *Lepus europaeus* | Oristà | 13-Feb-14 | Liver | GI.3P-GI.2 |
| LE15067 | PX672493 | *Lepus europaeus* | Les Avellanes i Santa Linya | 23-Nov-15 | Liver | GI.1bP-GI.2 |
| LE16032 | PX672494 | *Lepus europaeus* | Sant Joan de les Abadesses | 11-Oct-16 | Liver | GI.1bP-GI.2 |
| LE16034 | PX672495 | *Lepus europaeus* | Torregrossa | 22-Nov-16 | Liver | GI.4(p16)-GI.1bP-GI.2 |
| LE17005 | PX672496 | *Lepus europaeus* | Fígols i Alinyà | 05-Oct-17 | Liver | GI.4(p16)-GI.1bP-GI.2 |
| LE18001 | PX672497 | *Lepus europaeus* | Linyola | 12-Jul-18 | Liver | GI.4(p16)-GI.1bP-GI.2 |
| LE18006 | PX672498 | *Lepus europaeus* | La Granadella | 06-Nov-18 | Liver | GI.4(p16)-GI.1bP-GI.2 |
| LE18016 | PX672499 | *Lepus europaeus* | Aitona | 13-Nov-18 | Liver | GI.4(p16)-GI.1bP-GI.2 |
| LE18018 | PX672500 | *Lepus europaeus* | Pujalt | 28-Nov-18 | Liver | GI.4(p16)-GI.1bP-GI.2 |
| LE18020 | PX672501 | *Lepus europaeus* | Espluga de Francolí | 12-Dec-18 | Liver | GI.4(p16)-GI.1bP-GI.2 |
| LE19008 | PX672502 | *Lepus europaeus* | El Palau d’Anglesola | 27-Sep-19 | Liver | GI.4(p16)-GI.1bP-GI.2 |
| LE19013 | PX672503 | *Lepus europaeus* | Lleida | 27-Nov-19 | Liver | GI.4(p16)-GI.1bP-GI.2 |
| LE19014 | PX672504 | *Lepus europaeus* | Sarral | 11-Dec-19 | Liver | GI.4(p16)-GI.1bP-GI.2 |
| LE19015 | PX672505 | *Lepus europaeus* | Torres de Segre | 18-Dec-19 | Liver | GI.4(p16)-GI.1bP-GI.2 |
| LE20009 | PX625221, PX625234 | *Lepus europaeus* | Vimbodí i Poblet | 30-Sep-20 | Liver | GI.4P-GI.2 |
| LE21024 | PX625222, PX625235 | *Lepus europaeus* | Bordils | 26-Apr-21 | Liver | GI.4P-GI.2 |
| LE21037 | PX672506 | *Lepus europaeus* | Blancafort | 23-Nov-21 | Liver | GI.4P-GI.2 |
| LE22016 | PX672507 | *Lepus europaeus* | Llagostera | 21-Dec-22 | Liver | GI.4P-GI.2 |
| LE22017 | PX672508 | *Lepus europaeus* | Caldes de Malavella | 21-Dec-22 | Liver | GI.4P-GI.2 |
| LE23001 | PX625223, PX625236 | *Lepus europaeus* | Vilajuïga | 11-Dec-22 | Liver | GI.4-GI.1b-GI.2 |
| LG20001 | MZ203092 | *Lepus granatensis* | Sant Jaume dels Domenys | 20-Nov-20 | Liver | GI.4P-GI.2 |
| OC15002 | PX672509 | *Oryctolagus cuniculus* | Súria | 11-Mar-15 | Spleen | GI.1bP-GI.2 |
| OC15008 | PX672510 | *Oryctolagus cuniculus* | Palau-solità i Plegamans | 21-Dec-15 | Liver | GI.1bP-GI.2 |
| OC16001 | PX672511 | *Oryctolagus cuniculus* | Cassà de la Selva | 01-Mar-16 | Liver | GI.1bP-GI.2 |
| OC17020 | PX672512 | *Oryctolagus cuniculus* | Palau-solità i Plegamans | 03-May-20 | Liver | GI.1bP-GI.2 |
| OC17022 | PX672513 | *Oryctolagus cuniculus* | Palau-solità i Plegamans | 26-Sep-17 | Liver | GI.4(p16)-GI.1bP-GI.2 |
| OC17023 | PX672514 | *Oryctolagus cuniculus* | Palau-solità i Plegamans | 29-Sep-17 | Liver | GI.4(p16)-GI.1bP-GI.2 |
| OC17024 | PX672515 | *Oryctolagus cuniculus* | Palau-solità i Plegamans | 09-Oct-17 | Liver | GI.4(p16)-GI.1bP-GI.2 |
| OC17026 | PX672516 | *Oryctolagus cuniculus* | Palau-solità i Plegamans | 13-Oct-17 | Liver | GI.4(p16)-GI.1bP-GI.2 |
| OC18001 | PX672517 | *Oryctolagus cuniculus* | Palau-solità i Plegamans | 09-Feb-18 | Liver | GI.4(p16)-GI.1bP-GI.2 |
| OC18006 | PX672518 | *Oryctolagus cuniculus* | Marçà | 23-Apr-18 | Liver | GI.4P-GI.2 |
| OC18011 | PX672519 | *Oryctolagus cuniculus* | Palau-solità i Plegamans | 02-May-18 | Liver | GI.1bP-GI.2 |
| OC18012 | PX672520 | *Oryctolagus cuniculus* | Les Borges Blanques | 04-May-18 | Liver | GI.4(p16)-GI.1bP-GI.2 |
| OC18013 | PX672521 | *Oryctolagus cuniculus* | Flix | 04-May-18 | Liver | GI.4(p16)-GI.1bP-GI.2 |
| OC18014 | PX672522 | *Oryctolagus cuniculus* | Les Borges Blanques | 04-May-18 | Liver | GI.4(p16)-GI.1bP-GI.2 |
| OC18015 | PX672523 | *Oryctolagus cuniculus* | Palau-solità i Plegamans | 23-May-18 | Liver | GI.1bP-GI.2 |
| OC18017 | PX672524 | *Oryctolagus cuniculus* | Sant Feliu de Codines | 07-Jun-18 | Liver | GI.4(p16)-GI.1bP-GI.2 |
| OC19001 | PX672525 | *Oryctolagus cuniculus* | Vimbodí i Poblet | 11-Jan-19 | Liver | GI.4(p16)-GI.1bP-GI.2 |
| OC19003 | PX672526 | *Oryctolagus cuniculus* | Roses | 16-Jan-19 | Liver | GI.4(p16)-GI.1bP-GI.2 |
| OC19005 | PX672527 | *Oryctolagus cuniculus* | Granera | 20-Feb-19 | Spleen | GI.4(p16)-GI.1bP-GI.2 |
| OC19009 | PX672528 | *Oryctolagus cuniculus* | Oliola | 20-Dec-19 | Liver | GI.4(p16)-GI.1bP-GI.2 |
| OC20003 | PX672529 | *Oryctolagus cuniculus* | Santa Perpètua de Mogoda | 29-Jan-20 | Liver | GI.4P-GI.2 |
| OC20010 | PX625224, PX625237 | *Oryctolagus cuniculus* | Mollet del Vallès | 12-May-20 | Liver | GI.4-GI.2 |
| OC20017 | PX625225, PX625238 | *Oryctolagus cuniculus* | Mollet del Vallès | 23-Jun-20 | Liver | GI.4P-GI.2 |
| OC20044 | PX672530 | *Oryctolagus cuniculus* | Sant Jaume dels Domenys | 27-Nov-20 | Liver | GI.4P-GI.2 |
| OC21053 | PX672531 | *Oryctolagus cuniculus* | Torrebesses | 19-Feb-21 | Liver | GI.4(p16)-GI.1bP-GI.2 |
| OC21056 | PX672532 | *Oryctolagus cuniculus* | Caldes de Malavella | 10-Mar-21 | Liver | GI.4P-GI.2 |
| OC21057 | PX672533 | *Oryctolagus cuniculus* | Tiana | 10-Mar-21 | Liver | GI.4(p16)-GI.1bP-GI.2 |
| OC21060 | PX672534 | *Oryctolagus cuniculus* | Sant Jordi Desvalls | 26-Apr-21 | Liver | GI.4P-GI.2 |
| OC21095 | PX672535 | *Oryctolagus cuniculus* | Caldes de Malavella | 30-Sep-21 | Liver | GI.4P-GI.2 |
| OC22008 | PX625226, PX625239 | *Oryctolagus cuniculus* | Marçà | 26-Jan-22 | Liver | GI.4P-GI.2 |
| OC22038 | PX625227, PX625240 | *Oryctolagus cuniculus* | Lleida | 02-Aug-22 | Liver | GI.4P-GI.2 |
| OC22085 | PX672536 | *Oryctolagus cuniculus* | Godall | 18-Oct-22 | Liver | GI.4(p16)-GI.1bP-GI.2 |
| OC22100 | PX625228, PX625241 | *Oryctolagus cuniculus* | Brunyola i Sant Martí Sapresa | 25-Nov-22 | Liver | GI.4P-GI.2 |
| OC23001 | PX625229, PX625242 | *Oryctolagus cuniculus* | Maçanet de la Selva | 09-Jan-23 | Liver | GI.4P-GI.2 |
| OC23019 | PX672537 | *Oryctolagus cuniculus* | Oliola | 03-Feb-23 | Liver | GI.4P-GI.2 |
| OC23024 | PX625230, PX625243 | *Oryctolagus cuniculus* | Tortosa | 14-Mar-23 | Liver | GI.4(p16)-GI.1bP-GI.2 |
| OC23046 | PX672538 | *Oryctolagus cuniculus* | Barcelona | 18-Apr-23 | Liver | GI.4P-GI.2 |
| OC23099 | PX625231, PX625244 | *Oryctolagus cuniculus* | Freginals | 21-Nov-23 | Liver | GI.4(p16)-GI.1bP-GI.2 |
| OC24017 | PX672539 | *Oryctolagus cuniculus* | Castellar del Vallès | 16-Feb-24 | Liver | GI.4(p16)-GI.1bP-GI.2 |
| OC24023 | PX625232, PX625245 | *Oryctolagus cuniculus* | Torregrossa | 23-Feb-24 | Liver | GI.4(p16)-GI.1bP-GI.2 |
| OC24029 | PX672540 | *Oryctolagus cuniculus* | Tortosa | 11-Apr-24 | Liver | GI.4P-GI.2 |
| OC24034 | PX625248, PX625249 | *Oryctolagus cuniculus* | Viladecans | 28-Jun-24 | Liver | GI.4P-GI.2 |
| OC24052 | PX625233, PX625246 | *Oryctolagus cuniculus* | Mollet del Vallès | 23-Sep-24 | Liver | GI.4P-GI.2 |
| OC24060 | PX672541 | *Oryctolagus cuniculus* | Roses | 08-Nov-24 | Liver | GI.4P-GI.2 |

**LITERATURE CITED (not included in the manuscript)**

1. Oka T, Yokoyama M, Katayama K, et al (2009) Structural and biological constraints on diversity of regions immediately upstream of cleavage sites in calicivirus precursor proteins. Virology 394:119–129. <https://doi.org/10.1016/j.virol.2009.08.018>
2. McIntosh MT, Behan SC, Mohamed FM, et al (2007) A pandemic strain of calicivirus threatens rabbit industries in the Americas. Virol J 4:96. <https://doi.org/10.1186/1743-422X-4-96>
3. Forrester NL, Moss SR, Turner SL, et al (2008) Recombination in rabbit haemorrhagic disease virus: Possible impact on evolution and epidemiology. Virology 376:390–396. <https://doi.org/10.1016/j.virol.2008.03.023>
4. Nyström K, Le Gall-Reculé G, Grassi P, et al (2011) Histo-blood group antigens act as attachment factors of rabbit hemorrhagic disease virus infection in a virus strain-dependent manner. PLoS Pathog 7:e1002188. <https://doi.org/10.1371/journal.ppat.1002188>
5. Boga JA, Casais R, Marin MS, et al (1994) Molecular cloning, sequencing and expression in *Escherichia coli* of the capsid protein gene from rabbit haemorrhagic disease virus (Spanish isolate AST/89). J Gen Virol 75 ( Pt 9):2409–2413. <https://doi.org/10.1099/0022-1317-75-9-2409>
6. Lopes AM, Capucci L, Gavier-Widén D, et al (2014) Molecular evolution and antigenic variation of European brown hare syndrome virus (EBHSV). Virology 468–470:104–112. <https://doi.org/10.1016/j.virol.2014.08.002>
7. Forrester NL, Abubakr MI, Abu Elzein EME, et al (2006) Phylogenetic analysis of Rabbit haemorrhagic disease virus strains from the Arabian Peninsula: Did RHDV emerge simultaneously in Europe and Asia? Virology 344:277–282. <https://doi.org/10.1016/j.virol.2005.10.006>
8. Miao Q, Qi R, Veldkamp L, et al (2019) Immunogenicity in Rabbits of Virus-Like Particles from a Contemporary Rabbit Haemorrhagic Disease Virus Type 2 (GI.2/RHDV2/b) Isolated in The Netherlands. Viruses 11:553. <https://doi.org/10.3390/v11060553>
9. Lopes AM, Blanco-Aguiar J, Martín-Alonso A, et al (2018) Full genome sequences are key to disclose RHDV2 emergence in the Macaronesian islands. Virus Genes 54:1-4. <https://doi.org/10.1007/s11262-017-1523-2>
10. Strive T, Wright JD, Robinson AJ (2009) Identification and partial characterisation of a new lagovirus in Australian wild rabbits. Virology 384:97–105. <https://doi.org/10.1016/j.virol.2008.11.004>
11. Mahar JE, Nicholson L, Eden J-S, et al (2016) Benign Rabbit Caliciviruses Exhibit Evolutionary Dynamics Similar to Those of Their Virulent Relatives. J Virol 90:9317–9329. <https://doi.org/10.1128/jvi.01212-16>
12. Bergin IL, Wise AG, Bolin SR, et al (2009) Novel Calicivirus Identified in Rabbits, Michigan, USA. Emerg Infect Dis 15:1955–1962. <https://doi.org/10.3201/eid1512.090839>
13. Lopes AM, Marques S, Silva E, et al (2014) Detection of RHDV strains in the Iberian hare (*Lepus granatensis*): Earliest evidence of rabbit lagovirus cross-species infection. Vet Res 45:1–7. <https://doi.org/10.1186/s13567-014-0094-7>
14. Droillard C, Lemaitre E, Chatel M, et al (2018) First Complete Genome Sequence of a Hare Calicivirus Strain Isolated from *Lepus europaeus*. Microbiol Resour Announc 7:. <https://doi.org/10.1128/mra.01224-18>
